# Neurophysiological indices as predictive factors of clinical improvement

**DOI:** 10.1192/j.eurpsy.2025.2112

**Published:** 2025-08-26

**Authors:** L. Ghidetti, S. Costantini, S. Garofalo, M. Beghi, E. Ubaldini, S. Pecar, R. Raggini, R. P. Sant’Angelo

**Affiliations:** 1 Psychology, University of Bologna; 2 Mental Health; 3 Mental Health Department, Ausl Romagna, Cesena, Italy

## Abstract

**Introduction:**

In recent years, scientific literature has focused on the study of neurophysiological indices, including parameters derived from electroencephalography (EEG), electrocardiography (ECG), and electrodermal activity (EDA), in order to identify potential biomarkers that could be useful in the assessment, monitoring, and prevention of psychiatric disorders.

**Objectives:**

The aim of this work is to investigate whether some neurophysiological indices are able to predict the symptomatic improvement and the socio-relational functioning of people hospitalized in acute psychopathological conditions at the Psychiatric Unit of the Bufalini hospital in Cesena, in a transversal manner in regard to the diagnostic category and the symptomatic severity of the psychiatric disorder.

**Methods:**

To assess treatment outcomes from both a psychopathological and functional perspective, the Health of the Nation Outcome Scales (HoNOS) and Brief Psychiatric Rating Scale (BPRS) were administered at the start and end of hospitalization. Neurophysiological parameters were measured using the Rehacor-T device, which recorded frontocentral cerebral oscillations in Delta, Theta, Alpha, and Beta frequency bands, as well as heart rate and skin conductance. Data collection involved two phases: initially, participants viewed a calming landscape with eyes open, followed by a phase with eyes closed where they aimed to maintain tranquility. The Stroop test was then conducted. Statistical analyses were performed using JASP software.

**Results:**

The sample includes 112 patients (M = 57; F = 55), the average age of the participants is 44 years (SD = 16), while the average level of education is 11 years (SD =3).

The Beta frequency band in the eyes closed condition and the Delta frequency band in the eyes open condition show a positive regression with the changes in the HoNOS scale scores, indicating that an increase in Beta and Delta oscillations corresponds to an improvement in socio-relational functioning.

A positive regression was found between Delta oscillations in the Stroop test phase and changes in BPRS scores, suggesting that an increase in oscillations corresponds to an improvement in clinical symptoms.

**Conclusions:**

Although several autonomic abnormalities are known in psychiatric disorders, our results did not show any prognostic ability from the ECG and EDA indices recorded in the sample.

In conclusion, in line with what has already been demonstrated in the literature, it is possible to confirm that EEG indices can reflect the adaptive resources of the person affected by psychiatric disorders in terms of the possibility of responding to treatment.

Deepen the research in this field could lead to the identification of new and more specific biomarkers for the prevention, diagnosis and treatment of psychiatric disorders.

**Disclosure of Interest:**

None Declared

